# A Systematic Review of the Effects of Second-Eye Cataract Surgery on Motor Function

**DOI:** 10.3389/fragi.2022.866823

**Published:** 2022-06-22

**Authors:** William E. A. Sheppard, Dane McCarrick, Richard M. Wilkie, Rigmor C. Baraas, Rachel O. Coats

**Affiliations:** University of Leeds, Leeds, United Kingdom

**Keywords:** cataracat surgery, motor function, falls, aging, driving, activities of daily living, mobility

## Abstract

Cataract removal surgery is one of the most commonly performed surgical procedure in developed countries. The financial and staff resource cost that first-eye cataract surgery incurs, leads to restricted access to second-eye cataract surgery (SES) in some areas, including the United Kingdom. These restrictions have been imposed despite a lack of knowledge about the impact of not performing SES on visuo-motor function. To this end, a systematic literature review was carried out, with the aim of synthesising our present understanding of the effects of SES on motor function. Key terms were searched across four databases, PsycINFO, Medline, Web of Science, and CINAHL. Of the screened studies (*K* = 499) 13 met the eligibility criteria. The homogeneity between participants, study-design and outcome measures across these studies was not sufficient for meta-analyses and a narrative synthesis was carried out. The evidence from objective sources indicates a positive effect of SES on both mobility and fall rates, however, when considering self-report measures, the reduction in falls associated with SES becomes negligible. The evidence for any positive effect of SES on driving is also mixed, whereby SES was associated with improvements in simulated driving performance but was not associated with changes in driving behaviours measured through in vehicle monitoring. Self-report measures of driving performance also returned inconsistent results. Whilst SES appears to be associated with a general trend towards improved motor function, more evidence is needed to reach any firm conclusions and to best advise policy regarding access to SES in an ageing population.

**Systematic Review Registration:** https://osf.io/7hne6/, identifier INPLASY2020100042.

## 1 Introduction

Cataracts are a condition of the eye resulting in a clouding of the crystalline lens; a biconcave structure that works with the cornea to refract and focus light onto the retina and produce clear vision (Liu et al., 2017). Cataracts can occur in one eye (unilaterally) or both eyes (bilaterally). Cataracts can be classified as paediatric, those that present in early life; secondary to other causes, those due to an external event such as trauma or injury of the eye; and age-related. Age-related cataracts are the most common, with onset typically between the ages of 45–50 and are caused by oxidative stress of the lens (Liu et al., 2017). Regarding age-related cataracts there are three types: nuclear, cortical and posterior subcapsular (PSC); these are defined based on the location of the opacification within the eye. Nuclear cataracts occur centrally, cortical cataracts are often wedge-shaped and initially occur at the corner (cortex) of the eye protruding towards the centre, and PSC cataracts form as a plaque-like opacity in the axial posterior cortical layer (centrally, towards the bottom of the eye) (Liu et al., 2017).

Although cataracts can occur at any age, prevalence is far higher in older adults. A meta-analysis of 45 studies calculated pooled prevalence estimates (PPE; estimated using linear and random effects modelling) for the occurrence rate of cataract in different age groups. Prevalence of any type of cataract was found to be 3.01% (95% *CI*: 1.68–4.34) in 20–39 year-olds, 16.97% (95% *CI*: 11.36–22.57) in 40–59 year-olds and 54.38% (95% *CI*: 47.57–61.18) in over 60s (Hashemi et al., 2020). Cataracts are the leading cause of preventable blindness in over 50s, with an estimated 15.2 million cases of blindness caused by cataract globally in 2020, which is far higher than the 3.6 million cases of avoidable blindness caused by glaucoma, the second-highest cause (Steinmetz et al., 2021). With the trend towards an older population, the prevalence of cataracts in society only stands to increase, therefore, viable and affordable treatment options must be widely available.

Currently, surgery is the only proven treatment for cataracts and typically involves the removal and replacement of the clouded lens in a process known as phacoemulsification (Linebarger et al., 1999). The new, artificial lens often has some aspect of optical correction (i.e. 1 or two dioptres) meaning that the process, in the case of bilateral cataract removal surgery, often renders the patient spectacle free either at near or far distance (Bianchi, 2020). Due to the prevalence of cataracts and the efficacy and safety of phacoemulsification (Davis, 2016), surgery is the most commonly performed surgical procedure in the NHS (British National Health Service). Approximately, 400,000 procedures are performed each year at a cost exceeding £290,000,000 (National Institute for Health and Care Excellence, 2017; NHS Improvement, 2017).

In the United Kingdom and many other countries, it is commonplace to perform cataract removal surgery on the first eye (First-Eye Surgery; FES) followed by at least a 2-week interval before removing the cataract from the second eye (Second-Eye Surgery; SES). This process is referred to as delayed sequential bilateral cataract surgery (DSBCS) and is more commonly performed than the alternative surgical protocol of immediate sequential bilateral cataract surgery (ISBCS), where both cataracts are removed and replaced in the same surgical session (Spekreijse et al., 2020). DSBCS is often the preferred protocol to help mitigate the risk of post-operative complications such as bilateral infection (known as endophthalmitis) and refractive surprise (where cataract removal leaves the patient with an unexpected refractive error) (Kessel et al., 2016).

Delaying SES is associated with increased financial and resource (in terms of materials and staff-time) costs, ultimately reducing the capacity for patient care (Bhalla et al., 2021). To this end, access to SES has been limited by some Clinical Commissioning Groups (CCGs) through a ‘managed access’ approach based on arbitrary post-FES performance thresholds on a range of vision tests (Appleby, Devlin, Parkin, Buxton, and Chalkidou, 2009). This approach has come under some criticism, as in 2012, evidence emerged to suggest that nine-in-ten CCGs were restricting access to SES using criteria that reflected neither evidence nor clinical guidance (Coronini-Cronberg, Lee, Darzi, and Smith, 2012). This begs the question, are CCGs right to restrict access to SES and what consequences may this be having for patients?

In a meta-analysis of 13 studies including 705 participants (Frampton et al., 2014), SES was shown to generally improve visual function including contrast sensitivity (CS), an individual’s ability to detect images of low contrast (Mäntyjärvi and Laitinen, 2001), visual acuity (VA), the ability to read a standard test pattern at a specific distance (Messina and Evans, 2006), and stereopsis, the ability to gather information from retinal disparities (differences in the image received by either eye) (Rogers and Bradshaw, 1993; Orban et al., 2006). Findings such as this have been repeated more recently and have been shown to have a functional impact on the individual. For example, in a study investigating the impact of FES and SES on vision and the avoidance of driving in certain situations, known as driving self-regulation: mean binocular VA significantly improved from baseline (0.15 logMAR), after FES (0.08 logMAR) and SES (-0.02 logMAR), as did binocular CS (baseline 1.64 log units; post-FES 1.67 log units, post-SES 1.75 log units), stereopsis (baseline 2.14 log arcsecs; post-FES 2.31 log arcsecs, post-SES 1.96 log arcsecs), and the odds of self-regulation among participants (baseline *OR*: 1; post-FES *OR*: 0.3, 95% *CI*: 0.1–0.7, post-SES *OR*: 0.1, 95% *CI*: 0.1–0.4). Additionally, changes to binocular CS were associated with significantly decreased odds of self-regulation (*OR*: 0.02, 95% *CI*: 0.01–0.4).

However, clinical tests of vision, such as those reported in the previous paragraph, do not always track functional outcomes, for example, changes to stereopsis and VA were not associated with changes to fall risk following SES (Gia To et al., 2014). A well-functioning motor system is crucial to allow individuals to perform ADLs and maintain independence into older age, so a detailed understanding of how best to maintain this is crucial given the trend toward an ageing population (Seidler et al., 2010). One could argue that if changes to vision do not affect day-to-day life, they are clinically less significant. It is, therefore, crucial to also consider measures of functional motor performance, such as driving, falls and the ability of the individual to perform activities of daily living (ADLs; e.g. cooking, cleaning and maintaining personal hygiene), when considering the efficacy of SES. For example, in a study investigating the effects of cataract surgery on fall risk at baseline (pre-FES), post-FES and post-SES it was found that, compared to baseline, fall risk increased 114% post-FES and 34% post-SES, although no comparisons were made between the post-FES and post-SES data (Agramunt et al., 2018). This highlights a key shortfall in the body of work investigating the functional impact of SES; it is a common practice to use pre-FES scores of vision or motor function as the reference group for statistical analysis (Gia To et al., 2014; Meuleners et al., 2014; Agramunt et al., 2018; Feng et al., 2018). This practice interprets the separate effect of FES and SES difficult as no tests of difference have been performed between the post-FES and post-SES groups.

Even where direct comparisons between FES and SES are made, currently there is no systematic account/overview of how SES affects the motor system. For example, research into the effect of SES on mobility is inconsistent, whereby, there is evidence that SES reduce the time taken and the number of obstacles hit while navigating a course (Elliott et al., 2000), whereas, other studies have not found this effect (Elliott et al., 1997). A similar pattern emerges in the driving literature whereby some studies predict a positive effect of SES on driving behaviours (Meuleners et al., 2021) and others find no significant effect (Lundstrom et al., 2006).

Therefore, there seems a legitimate need to investigate and synthesize our present understanding of the impact of SES on the motor system through carrying out a systematic review of the literature. This will include identifying motor functions that stand to benefit most from SES as well as identifying areas that present mixed results that require future research. Having completed the systematic review, the results will be used to inform a schedule of experimental work investigating the impact of cataract and (artificially) unilaterally degraded vision on motor function and how this is related to changes of vision.

## 2 Methods

This review was pre-registered with PROSPERO (CRD42021231856), this pre-registration is available on the Open Science Framework (DOI: 10.17605/OSF.IO/7HNE6, https://osf.io/7hne6/?view_only=f2d8d3056bba40ef939447608942b92f).

### 2.1 Eligibility Criteria

Studies were included if they: 1) were empirical studies generating novel data; 2) met the following PICOS criteria: P (Population): age-related cataract patients; I (Intervention/Exposure): second-eye cataract removal surgery; C (Comparison): first-eye surgery and/or waiting for second-eye surgery; O (Outcome): any motor measure; S (Study design): any empirical design. Studies were excluded if: 1) participants were under the age of 18; 2) cataracts were secondary to other causes such as trauma or illness; 3) participants had motor co-morbidities such as Parkinson’s disease, or patients had a history of cataract removal surgery in the operative eye (i.e. they were having a second or third cataract removed from the same eye); 4) they were a review study (i.e., systematic review or meta-analysis); 5) they were not in English; 6) they did not use human samples.

### 2.2 Search Strategy

Four databases were searched to maximize search sensitivity (Montori et al., 2005): PsycINFO (1806–present) and Medline (1806–present) via OVID, Web of Science (1900–present), and CINAHL (1960-present) using EBSCO. The search was last conducted on 23rd October 2020. The full search list of search terms can be found in *Supplementary Text 1*. However, for brevity, studies were included if they contained the word/phrase: cataract; surgery, removal or extraction; first-eye or second-eye; some reference to motor function or specific motor tasks such as driving or mobility.

First, titles, abstracts, and full-text screening were completed by the first author. Then, the second author independently screened the titles and abstracts of all (100%) of studies (*K* = 497, *Cohen’s kappa* = 1.00). Any study identified as potentially eligible at the abstract screening stage was progressed to full-text screening (*K* = 43). Second, the first author independently screened all full-texts (*K* = 43), before all (100%) of the of full-texts were double screened by the second author (*Cohen’s kappa* = 1.00). Across the sets of double-screened studies at each stage of the screening and data extraction process, the secondary coder did not identify any eligible studies missed by the primary coder.

### 2.3 Data Extraction and Coding

The subsequent data were extracted and coded for each study: lead author name, publication year, country, study design (RCT, longitudinal, cross-sectional etc.), measurement points (stage of surgery i.e. pre-FES, post-FES, post-SES, time since surgery etc.) for motor outcomes (i.e. driving performance such as crash rates, mobility scores and fall incidences, questionnaire responses e.g., VF-14, Catquest-95), statistical analysis (i.e., ANOVA, Incidence Risk Ratio, Generalised Estimating Equations) participant characteristics: age, percentage female, and the number of participants included in analysis and attrition (across the entire study).

Quality assurance was then carried out using the Mixed Methods Appraisal Tool 2018 (MMAT) (Hong et al., 2019). This is a tool designed to appraise the quality of studies using different designs including qualitative research, randomized controlled trials, non-randomized studies, quantitative descriptive studies, and mixed methods studies, so was deemed appropriate for the present review due to the heterogeneous nature of the included studies. It has been used previously and validated in similar healthcare reviews (Catsaros and Wendland, 2020; Lin et al., 2020; Pimentel et al., 2020; Wu et al., 2020). We approached data extraction in two phases to minimise the possibility of coding errors. The first phase was piloted on 1 (9.09%) of the studies in a “training phase”. For this piloted study, the coding for all measures was checked by a second reviewer. Inter-rater agreement levels were classified as perfect for MMAT items relating to screening (*Cohen’s kappa* = 1.00) and quality assessment (*Cohen’s kappa* = 1.00) (Landis and Koch, 1977). Second, we operated a “validation phase” whereby data for all studies were first extracted by a primary coder before all studies were independently assessed by a second coder. For this phase, the agreement between coders was almost perfect across all MMAT items (*Cohen’s kappa* = 0.88) (Landis and Koch, 1977). In all cases, if either coder was in any doubt, the study authors were contacted for additional clarification before deciding upon eligibility (*K* = 6).

### 2.4 Data Synthesis

Due to the highly heterogeneous nature of study designs and outcomes measures included in the sample, as well as the small size of the sample, the data were deemed unsuitable for meta-analyses or other advanced statistical techniques (Higgins et al., 2021). Therefore, no further statistical analysis was planned, and a narrative synthesis was conducted. This demonstrates a key issue identified in the present work, that the body of research upon which medical decisions are being based lacks a consistent approach, indicating the need for a unified approach to SES research. In the case where a study contained multiple measures of motor function, all will be reported.

Broadly, the narrative synthesis consisted of two branches: primary outcome measures including experimentally collected changes to motor function due to SES i.e., driving performance such as crash rates, mobility scores and fall incidences; and secondary outcomes including self-report measures of quality of life incorporating items regarding motor function i.e., VF-14, Catquest-95. These branches will be further split by whether data were collected/analysed between- or within-participants due to the increased error variance associated with between-participants designs (Thompson and Campbell, 2004).

## 3 Results

**FIGURE 1:**
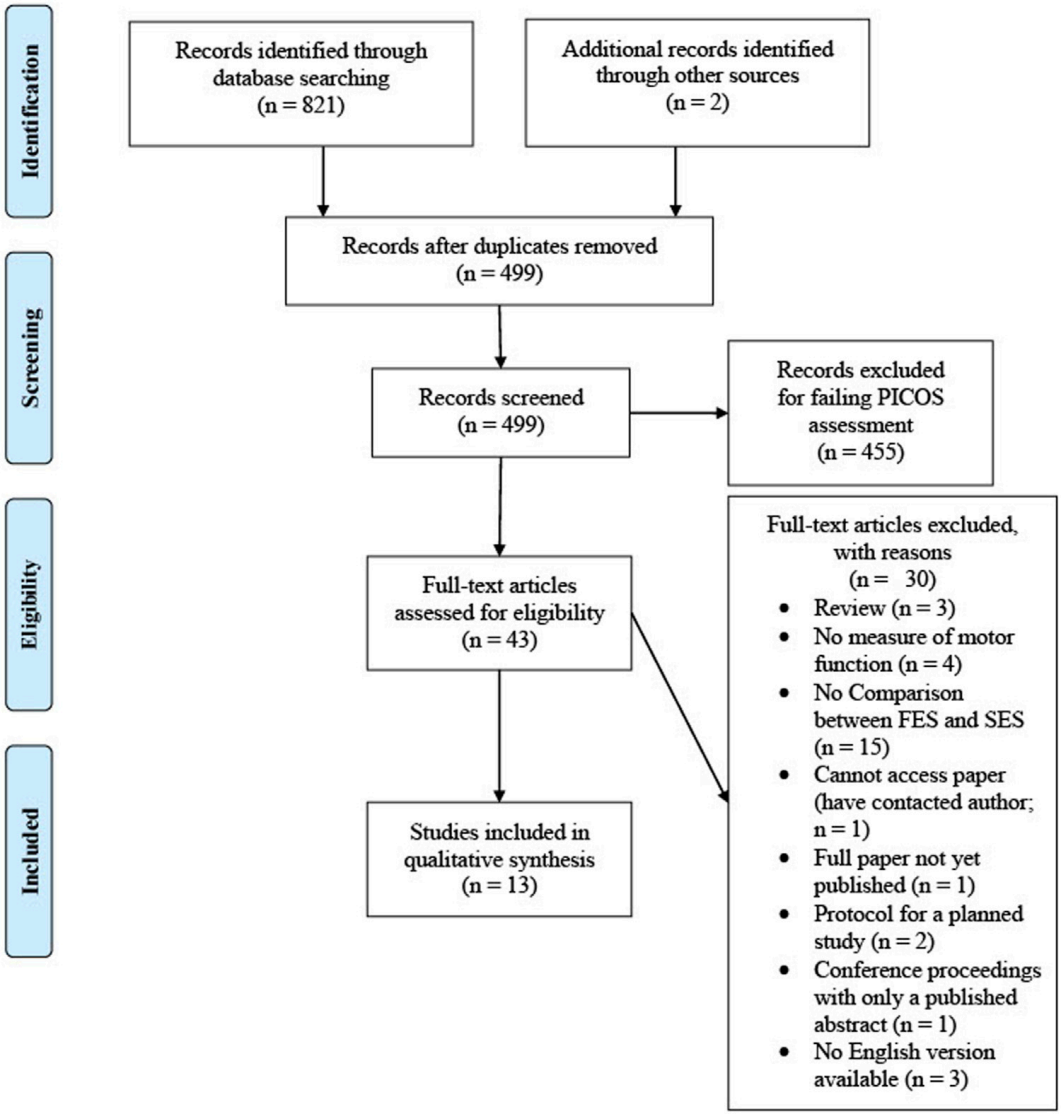
Prisma flow diagram.

A systematic review of the literature (carried out as per Search Strategy, page 4) returned 821 records, which were reduced to 497 after duplicates were removed. Having screened the titles and abstracts of these records, 41 were deemed to be of potential interest, the full texts were obtained and screened (as per inclusion/exclusion criteria, page 7). Of these 41 records, 11 were deemed to assess the separate impact of SES on motor function (see *Figure 1*) (Agramunt et al., 2018; Akman et al., 2019; Castells et al., 1999; Elliott et al., 2000; Feng et al., 2018; Foss et al., 2006; Gia To et al., 2014; Lee et al., 2013; Lundstrom et al., 2006; Meuleners et al., 2014, 2019). Two papers were also included that were found outside of the search (Elliott et al., 1997; Meuleners et al., 2021), giving a total of 13 papers to be included in the synthesis. The characteristics of these studies are shown in Table 1 and the quality assessment (assessed using the MMAT (Hong et al., 2019)) is shown in Table 2.

**TABLE 1:** Study characteristics. * representants quality assessment score out of five.

Author, date (quality)	Design	Location	Comparison	Measurement point (time pre/post-surgery)	Between− or within-subjects	Objective motor outcomes	Subjective motor outcomes	Participant characteristics	participants included:k, mean age (sd), % Female	Attrition (across entire study)
Agramunt et al.,2018 (****)	Prospective cohort study	Australia	Post−FES and Post−SES vs. Pre−FES	Within 1 month pre−FES, 1–3 months post−FES, at least 1 month post−SES	Within-subjects	In−vehicle monitoring	Driving Habits Questionnaire, travel diary	Convenience sample of current drivers with bilateral cataract	55,73.3 (7.8),54.5%	50.5
Akman et al., 2019 (*****)	Prospective noncomparative case-series	Turkey	Post−FES vs. post−SES	Pre−FES (not specified), 3 months post−FES and 3 months post−SES	Within-subjects	N/A	VF−14 QoL questionnaire	Convenience sample of bilateral cataract patients fitted witha trifocal IOL	48,65.1 (8.4),64.6%	0
Castells et al., 1999 (***)	Comparative case-series	Spain	Post−FES vs. post−SES	Pre−FES (not specified), 4 months post−FES or post−SES	Between-subjects	N/A	Spanish VF−14 QoL questionnaire	Convenience sample of patients scheduled for cataract surgery	315, FES:249,69.8 (11.3),53.0% SES:66,70.1 (10.1),62.1%	21.8
Elliott et al., 1997 (****)	Comparative case-series	Canada	Control vs. Post−FES vs. Post−SES	Pre− and post-operative. SES: 8.7 weeks between session, FES: 8.9 weeks. Controls: 4.2 weeks	Between-subjects	Obstacle avoidance, mobility orientation	ADVS	Convenience sample of patients scheduled to have cataract surgery within the next month	26, SES:10,67.4 (8.3), FES:6,72.1 (6.2), Control:10,69.1 (4.3), % Female not reported	36.0
Elliott et al., 2000 (***)	Comparative case-series	Canada	Control vs. Post−FES vs. post−SES	Pre− and post-operative. SES: 10.8 (6.0) weeks between session, FES12.2 (5.8) weeks. Controls 13.6 (4.6) weeks	Between-subjects	Obstacle avoidance, mobility orientation	ADVS	Convenience sample of patients scheduled to have cataract surgery within the next month	68, SES:25,71.3 (9.5), FES:18,74.3 (6.1), Control:25,70.6 (4.6), % Female not reported	28.8
Feng et al.,2018 (****)	Prospective cohort study	Australia	Post−FES and post−SES vs. Pre−FES	Within1 month pre−FES, between FES and SES, at least 1 month post−SES, Further follow-up 4–6 months post−SES	Within-subjects	N/A	Falls diary, Active Australia Survey	Convenience sample of patients scheduled for cataract surgery	55,73.3 (7.7),54.6%	0
Foss et al., 2006 (****)	RCT	United Kingdom	Post−FES vs. post−SES	Falls: 3 &9 months post-randomisation, ADLs and visual disability: 6 &12 months post-randomisation	Between-subjects	N/A	Falls diary, Barthel Index, VF−14	Convenience sample of women over70s waiting for SES	239, Expedited:120,79.2 (median),100%. Routine:119,79.9 (median),100%	8.8
Gia To et al., 2014 (****)	Prospective cohort study	Vietnam	Post−FES or post−SES vs. Pre−FES	Week prior to FES, 1–3 months post−FES/SES, 1 year post−FES	Between− subjects	N/A	Falls diary	Convenience sample of independently living patients scheduled for cataract surgery	413,66.6 (7.9),64%	41.2
Lee et al., 2013 (*****)	Prospective, population based study	USA	No surgery vs. Post−FES vs. post−SES	Baseline, 2 years post-baseline	Within− subjects	Timed 4m walk, stair ascent/descent, get-up-an-go test	ADVS	Sample of Salisbury Eye Evaluation enrolled participants	1739, No surgery:1630,71.6 (median),57%. Post−FES:90,76.1,52%. Post−SES:29,73.0,69%	0
Lundstrom et al., 2006 (***)	RCT	Sweden	Same day bilateral cataract extraction vs. post−FES	Baseline (pre−FES, not specified),2 months post first surgery,4 months post-last surgery	Between− subjects	N/A	Catquest	Convenience sample of patients scheduled for bilateral cataract surgery	96, ISBCS:50,72.5 (SD not reported),54.0%. DSBCS:46,72.5 (SD not reported),54.3	8.3
Meuleners et al., 2019 (****)	Prospective cohort study	Australia	Post−FES or post−SES vs. Pre−FES	Month pre−FES, post−FES (59.7 days, sd=41.3), post−SES (111.4 days, sd=40.2)	Within− subjects	N/A	Active Australia Survey	Convenience sample of patients scheduled for bilateral cataract surgery	55,73.3 (7.8),54%	0
Meuleners et al., 2014 (*****)	Retrospective cohort study	Australia	Post−FES and post−SES vs. pre-fes	2 years pre−FES, between FES and FES,2 years post−SES	Within− subjects	Injurious fall data extracted from medical records	N/A	All over60s who underwent BC surgery during the study period	28,396, Mean age not reported,58.4%	
Meuleners et al., 2021 (*****)	Prospective cohort study	Australia	Pre−FES vs. Post−FES vs. Post−SES	1 month pre−FES, 1–3 months post FES, 1+ months post−SES	Within− subjects	Simulated driving performance	N/A	Convenience sample of patients scheduled for bilateral cataract surgery	44,73.2 (8.3),47.7%	0

**TABLE 2:** MMAT Quality assessment table (Hong et al., 2018).

Author, Date	Criteria from the mixed methods appraisal tool																									
	1.1	1.2	1.3	1.4	1.5	2.1	2.2	2.3	2.4	2.5	3.1	3.2	3.3	3.4	3.5	4.1	4.2	4.3	4.4	4.5	5.1	5.2	5.3	5.4	5.5	Quality
Agramunt et al., 2018											∗	∗	-	∗	∗											∗∗∗∗
Akman et al. (2019)																∗	∗	∗	∗	∗						∗∗∗∗∗
Castells et al. (1999)											∗	-	-	∗	∗											∗∗∗
Elliott et al. (2000)											∗	∗	-	∗	-											∗∗∗
Feng et al., 2018											∗	∗	-	∗	∗											∗∗∗∗
Foss et al. (2006)						∗	∗	∗	-	∗																∗∗∗∗
Gia To et al. (2014)											∗	∗	-	∗	∗											∗∗∗∗
Lee et al. (2013)											∗	∗	∗	∗	∗											∗∗∗∗∗
Lundstrom et al. (2006)						-	∗	∗	-	∗																∗∗∗
Meuleners et al. (2019)											∗	∗	∗	-	∗											∗∗∗∗
Meuleners et al. (2014)											∗	∗	∗	∗	∗											∗∗∗∗∗

From the final selection of 13 papers: 6 collected objective measures of motor performance, including in-vehicle trip monitoring (Agramunt et al., 2018); obstacle avoidance and mobility orientation (Elliott et al., 1997, 2000); timed 4 m walk, stair ascent/descent, get-up-and-go test (Lee et al., 2013); the extraction of injurious fall data from medical records (Meuleners et al., 2014); and simulated driving performance (Meuleners et al., 2021). From these 6 papers: 4 employed a within-subjects design (Agramunt et al., 2018; Lee et al., 2013; Meuleners et al., 2014, 2021) and two used a between-subjects design (Elliott et al., 1997, 2000). Of the 13 papers, 11 collected subjective measures of motor function including the VF-14 QoL questionnaire (Castells et al., 1999; Foss et al., 2006; Akman et al., 2019); falls diary (Foss et al., 2006; Gia To et al., 2014; Feng et al., 2018); Active Australia Survey (Feng et al., 2018; Meuleners et al., 2019); Barthel Index (Foss et al., 2006); Activities of Daily Vision Scale (ADVS) (Elliott et al., 1997, 2000; Lee et al., 2013); and Catquest (Lundstrom et al., 2006). From these 11 papers: 5 employed a within-subjects design (Lee et al., 2013; Agramunt et al., 2018; Feng et al., 2018; Akman et al., 2019; Meuleners et al., 2019) and 6 used a between-subjects design (Elliott et al., 1997, 2000; Castells et al., 1999; Foss et al., 2006; Lundstrom et al., 2006; Gia To et al., 2014).

The original plan, as per the pre-registration, had been to sub-divide the synthesis by study design, however, this would have made the groups too small to draw any meaningful comparisons. Therefore, study design will be stated for each study, but this will not be used for the synthesis. Objective measures will be reported first, before subjective measures; furthermore, studies using within-subjects will be reported before between-subjects. This is due to a desire to report the strongest evidence, in terms of experimental power, first, before looking to confirm these effects with the ‘weaker’ evidence.

### 3.1 Objective Measures of Motor Function

#### 3.1.1 Within-Subjects (Agramunt et al., 2018; Lee et al., 2013; Meuleners et al., 2014; 2021)

Three motor functions were studied using objective, within-subjects methods: driving, falls and general motor function.

In a prospective cohort study, Agramunt and colleagues (2018) investigated changes in driver self-regulation i.e., changes in driving behaviours to avoid certain situations. They used a hybrid method whereby participants reported the likelihood of themselves driving in different situations. This was measured pre-FES, post-FES, and post-SES. These data were then cross-referenced with a trip diary and data from an in-vehicle monitoring system that recorded the time, distances and speed travelled. These data will, therefore, be considered an objective measure of motor performance and not discussed in the subjective measures section. Based on these data, participants were classified as a self-regulator or not a self-regulator. To make this a more conservative measure of driver self-regulation, and to avoid overstating the benefits of cataract removal surgery, when these data sources (participant reported likelihood vs. in-vehicle monitoring) were in disagreement, participants were classified as a non-self-regulator. Regarding specific driving situations, cataract surgery significantly predicted reductions in the percentage of participants classified as self-regulators in night driving (pre-FES 37.0%; post-FES 21.7%; post-SES 10.9%) and driving in heavy traffic (pre-FES 12.5%; post-FES 8.3%; post-SES 2.1%). Cataract surgery also predicted a significant reduction in overall rates of self-regulation. Generalized Estimating Equations (GEE) controlling for a range of confounders including cognitive status, age group (55–64/65–74/75 + years), gender, marital status, retirement status and the number of comorbidities found that the odds of being a self-regulator dropped 70% post-FES (*OR* = 0.3, 95% *CI* = 0.1–0.7) and 90% after SES (*OR =* 0.1, 95% *CI* = 0.1–0.4) compared to pre-FES. No direct comparison is made between post-FES and post-SES levels of self-regulation, however, despite the apparent reduction in self-regulation following SES, the overlap of the *CI*s presented in the analysis suggests that there is no significant effect of SES on driver self-regulation. The age of the participants did not significantly predict self-regulation and the time between surgeries is not reported.

Further research into the effects of cataract surgery on driving performance was carried out by Meuleners and colleagues (2021) in a prospective cohort study of simulated driving performance. This study was tightly controlled, excluding participants under 55, those who did not drive regularly, and those who scored poorly on the MMSE (Mini-Mental State Exam, a measure of cognitive function). Furthermore, driving performance was assessed in a simulated environment, thus, giving the researchers ultimate control over the visual stimuli. The simulated environment was similar to 10 km of typical Western Australian Road–where the study was performed. Participants were scored on non-compliance with the speed limit, speed variation, lane keeping and crashes/near crashes. A separate GEE Poisson or linear regression was calculated for each outcome variable including a large number of confounding variables including age (55–69/70–84/85 + years), gender and comorbidities. SES was found to reduce crash/near crash rates by 47% (*IRR* = 0.53, *CIs* = 0.35, 0.78, *p* < 0.001) compared to pre-FES levels and post-hoc analysis revealed that crash/near crash rates were also significantly lower when compared with post-FES levels (*p* = 0.002). Participants aged 70 and over were found to have significantly more instances of crashes/near crashes when compared with participants under the age of 70. The amount of time speeding did not significantly change following FES compared to pre-FES levels (*p* = 0.426), however, post-SES this effect was significant (*mean reduction* = 0.14 min, *p* = 0.002). Post-hoc testing found no significant difference between post-FES and post-SES levels of speeding (*p* = 0.52). No effect of age was found for non-compliance to speed limits. The time between FES and the driving-simulator assessment ranged from 9–417 days (*mean* = 99.6, *sd* = 73.7) and the time delay between SES and the driving-simulator assessment ranged from 29–238 days (*mean* = 112.3, *sd* = 40.6), the potential effect of this delay was not controlled for.

Meuleners and colleagues (2014) found further evidence for the positive effect of SES in a retrospective cohort study investigating fall risk. The medical records of over 28,000 bilateral cataract patients in Western Australia covering the 2 years pre-FES, the period between FES and SES and the 2 years post-SES were explored. They found that fall risk increased 114% post-FES (risk ratio [*RR*] = 2.14, 95% *CI* = 1.82–2.51) compared to pre-FES. This risk fell to an increase of 34% post-SES (*RR* = 1.34, 95% *CI =* 1.16–1.55) compared to pre-FES. Similarly, to the work presented by Agramunt and colleagues (2018), there are no direct comparisons made between post-FES and post-SES risk of falls, however, the confidence intervals presented above do not overlap, suggesting that SES significantly reduces fall risk, although not to pre-FES levels. The authors note that whilst the risk of falls increases post-SES compared to pre-FES, timely SES is still necessary to minimise fall risk. This is due to the strong relationship between increased age and increased fall risk, supported by the finding that participants over the age of 85 were 7 times more likely to fall during the study period compared to 60 to 65 year-olds (95% *CI* = 3.93–11.93). The time between FES and SES is not reported.

The trend towards reduced mobility in older adults is echoed in a large, prospective, population-based study including approximately 1700 participants (Lee et al., 2013). During the study period, 1,620 participants had no cataract surgery, 90 had unilateral surgery and 29 had bilateral cataract surgery. These participants all took part in a mobility assessment. After controlling for baseline performance, sex, age, comorbidities and other covariates, overall mobility declined between time points in all groups. The unilateral surgery group declined significantly more when compared with the no surgery group (*z* = -0.18, *p* < 0.05), whereas the bilateral surgery group trended towards improving compared with the no surgery group (*z* = 0.18, *p* > 0.05). These results do, however, demonstrate the benefits of timely SES over and above FES only. This is particularly relevant in the present example, as it is not clear in the original manuscript whether all participants in the unilateral surgery group had bilateral cataracts, suggesting that the positive effects of SES are a conservative estimate of what individuals stand to gain in terms of motor function from SES. Conversely, the lack of clarity also makes interpretating the results more challenging as we cannot be sure of the characteristics of the control, no surgery group. The time interval between surgeries is not reported.

##### 3.1.1.1 Conclusion

From the data presented here collected via within-subjects designs with objective measures of motor performance; we can conclude that SES has no significant effect on rates of driver self-regulation (Agramunt et al., 2018), however, SES does appear to significantly reduce fall rates (Meuleners et al., 2014) and improve mobility and simulated driving performance (Lee et al., 2013; Meuleners et al., 2021), when compared to post-FES levels. However, it is difficult to draw direct comparisons between these results due to the heterogeneity of research design between the studies, as well as a lack of direct comparisons between post-FES and post-SES groups.

#### 3.1.2 Between-Subjects (Elliott et al., 1997; 2000)

In a comparative case series, Elliott and colleagues 1997) investigated differences in obstacle avoidance and mobility orientation, the ability to navigate paths containing a variety of obstacles, in groups of participants before and after undergoing FES and SES. A group of healthy age match controls were also tested. There were no significant differences in age between the groups and the time between testing was also similar between the surgery groups and shorter in the control group. No significant effect of SES was found, however, the groups were small (*N* = ∼10) and post-SES scores were largely comparable to those of the healthy control group. In a follow-up study with larger groups (*N* = ∼20), Elliott and colleagues (2000) found that SES was associated with significant improvements in mobility orientation (which returned to the same level as the controls), objects collided with, and time taken to complete a walked path. The number of hits when stepping over a low target was also reduced. To ensure that these results were not due to practice i.e., performing the tasks before surgery and again after, the age-matched controls were tested twice. No significant effect of test-retest was found on any measures, apart from binocular VA, suggesting that the differences in performance in the SES group can be attributed to the benefits of the surgery. As per Elliott and colleagues (1997), there were no significant differences in age between the groups and the time between testing was also similar between the surgery groups and shorter in the control group.

##### 3.1.2.1 Conclusion

These results provide compelling and robust evidence that SES improves the ability of individuals to navigate their environment. Evidence for this effect can be seen as mobility orientation returns to similar levels as healthy age-matched controls post-SES, an effect not seen post-FES (Elliott et al., 1997; 2000). We can be confident in the robustness of these findings due to the similar ages and inter-test intervals of the surgery groups. This also supports the findings of Meuleners and colleagues (2014) and Lee and colleagues (2000), suggesting that the positive effects of SES can be seen both between- and within-participants.

### 3.2 Subjective Measures of Motor Function

The measures previously discussed were objective measures of motor performance, that is to say, that they are experimentally controlled and collected by a third party (the researcher) and that they are free from social desirability and self-report bias (Yang et al., 2016). However, these can be criticised for failing to capture information on the more ‘human’ side of changes to motor function such as the subjective ongoing experience of the participant, as well as their perceived competencies and ability to perform ADLs (Stuifbergen et al., 2014; Nielsen et al., 2016). This is where subjective/self-report measures of motor function are particularly useful and have been linked to long-term health outcomes (Osoba, 2011).

#### 3.2.1 Within-Subjects (Lee et al., 2013; Agramunt et al., 2018; Feng et al., 2018; Akman et al., 2019; Meuleners et al., 2019)

In a prospective cohort study, Akman and colleagues (2019) investigated the changes in functional vision in participants pre- and post-SES using the VF-14 QoL questionnaire. Post-SES, participants reported significant improvements in sewing and fine-handwork (*p* = 0.02) as well as reduced difficulties using a personal computer (*p* = 0.03). However, significant effects were not reported on any of the 12 other subscales (see Table 3). Participants were tested 3 months post-FES and 3 months post-SES with at least a 3 months gap between surgeries, however, the length of the gap between surgeries is not reported. No control for the effect of age is reported. The effects of cataract surgery on self-reported driving difficulties were measured using the Activities of Daily Vision Scale (ADVS) in a large, prospective, population-based study by Lee et al. (2013). After controlling for baseline performance, sex, age, comorbidities and other covariates, compared to participants who did not undergo cataract surgery, those who underwent unilateral surgery reported significant impairment in both day (*z* = −9.0, *p* < 0.05) and night driving (*z* = −8.4, *p* < 0.05), whereas those who underwent bilateral cataract surgery reported a non-significant impairment in day driving (*z* = −1.0, *p* > 0.05) and a non-significant improvement in night driving (*z* = 5.0, *p* > 0.05). The time interval between surgeries is not reported.

**TABLE 3:** VF-14 quality of life values.

Reading Small Print
Reading a newspaper or a book
Reading a large-print book or numbers on a telephone
Recognizing people when they are close to you
Seeing steps, stairs, or curbs
Reading traffic, street, or store signs
Doing fine handwork like sewing
Writing checks or filling out forms
Playing games such as bingo, dominos, card games, mahjong
Taking part in sports like bowling, handball, tennis, golf
Cooking
Watching television
Driving during the day
Driving at night
Recognizing people from a distance
Using a personal computer
Shaving, styling hair, or putting on makeup
Difficulty in going out to see movies, theater, plays, sports events

The effect of cataract surgery on fall rate was investigated in older adults using a fall diary in a prospective cohort study (Feng et al., 2018). When controlling for potential confounds such as age and the time interval between surgeries, when compared to pre-FES, the number of falls fell 54% following FES (*IRR* = 0.458, 95% *CI* = 0.215–0.974, *p* = 0.043) and 73% following SES (*IRR* = 0.268, 95% *CI* = 0.114–0.628, *p* = 0.002). Although no direct comparisons are made between the rate of falls following FES and SES, the *CI*s overlap, suggesting that there is no significant further reduction in falls following SES. However, there does seem to be a trend towards a reduction in falls post-SES compared with post-FES. Feng and colleagues (2018) also collected data on the amount of time each participant spent exercising per week using the Active Australia Survey (AAS) at each time point. Participants were classified as doing ‘sufficient’ exercise if they exercised for 30 min on at least 5 days per week or completed a total of 150 min of activity each week. At baseline, 56.4% of participants were deemed to complete sufficient exercise, compared to 50.9% post-FES and 56.4% post-SES. No inferential statistics are performed on these data. Despite this, there again seems to be a trend towards improved levels of physical activity following SES compared to post-FES, although this is only returning to baseline levels. This inference is supported by the work of Meuleners and colleagues (2019), who in a prospective cohort study found an increase in physical activity of 32 min per week post-SES compared to post-FES (*p* = 0.02; as measured by the AAS). This effect was robust when controlling for a range of confounding variables such as age, inter-surgery interval and comorbidities.

##### 3.2.1.1 Conclusion

From the data presented here, it appears that SES significantly improves the ability to perform specific motor tasks i.e. sewing and fine handwork and using a personal computer (Akman et al., 2019); as well as having an apparent, but non-significant, positive effect on both day and night driving (Lee et al., 2013). Again, we must be cautious in our interpretation of Lee and colleagues (2013) result, due to the lack of clarity in the characteristics and cataract status of the control, no surgery, group.

As per the evidence collected from an objective, within-subjects study of fall risk in older adults (Meuleners et al., 2014), Feng and colleagues (2018) demonstrated a trend toward reduced fall risk post-SES compared with post-FES, although there was no direct comparison between the post-FES and post-SES groups. This was coupled with an increase in self-reported physical leisure activity, a finding that was replicated by Meuleners and colleagues (2019), both of which were measured using the AAS.

#### 3.2.2 Between-Subjects (Elliott et al., 1997, 2000; Castells et al., 1999; Foss et al., 2006; Lundstrom et al., 2006; Gia To et al., 2014)

In a comparative case series comparing visual function post-FSE and post-SES using the VF-14 questionnaire, there was no significant difference on any subscale between the groups. The groups were matched by age, gender, comorbidities and pre-FES VA, however, the time gap between surgeries is not reported (Castells et al., 1999). The same measure of visual function was used by Foss and colleagues (2006) in an RCT. The authors report a significant improvement in the SES group relative to the FES group (*mean difference* = 7.5, *p* < 0.0005), however, they do not report the results of the subscales and as the VF-14 contains items regarding visual function. Meaning that the unique effect of SES on motor function cannot be determined. In this study, participants also completed the Barthel Index, a measure of the ability to complete activities of daily living such as feeding bathing and dressing. No significant difference between the groups (*mean difference* = -0.1, *p* = 0.61) was reported. The groups were well matched, and measurements were taken at 3, 6, 9 and 12 months post-randomisation, with baseline vision assessments carried out at 4 weeks post-FES. The overall time elapsed since FES for each measurement point is not reported. During the study period participants also completed a fall diary. Despite the SES group falling less than the FES group, this effect was not significant (*RR*: 0.68, 95% *CI* 0.39, 1.19, *p* = 0.18). The impact of FES and SES on fall risk was also investigated in a prospective cohort study by Gia To and colleagues (2014). Using a multilevel modelling approach, controlling for within-subject, level 2 factors including age, comorbidities and clinical vision, compared to pre-FES, rates of falling fell 78% (*IRR* 0.22, 95% *CI* 0.06–0.77; *p* = 0.018) post-FES and 83% (*IRR* 0.17, 95% *CI* 0.04–0.69; *p* = 0.01) post-SES. Whilst no direct comparison was made between the groups, the overlap of the confidence intervals suggests that SES does not have a significant impact on fall rates beyond that achieved by FES.

The link between cataract surgery and changes to self-reported disability were further investigated in an RCT comparing the effects of ISBCS and DSBCS in age-matched groups. Participants self-administered the Catquest, a measure of disability including measures of ability to perform ADLs, cataract symptoms, satisfaction with vision and driving. Patients completed the Catquest pre-operatively, at 2 months post-operatively (when the ISBCS group have had both cataracts removed and DSBCS have had one cataract removed) and again at 4 months post-operatively (when both groups have had both cataracts removed) (Lundstrom et al., 2006). Before surgery, there was no significant difference in total disability score (*p* = 0.966) or car driving (*p* = 0.711); at 2 months the ISBCS group had a significantly lower (improved) overall disability score (*p* < 0.001) and a non-significantly improved car driving score (*p* = 0.053); at 4 months these group differences were no longer apparent for the overall disability score (*p* = 0.481) or car driving (*p* = 0.254). This demonstrates that SES has a positive impact of SES on overall disability and a trend towards improved driving. However, the overall disability score also includes measures of satisfaction with vision and cataract symptoms so cannot be considered a pure measure of motor function.

Finally, the effect of cataract surgery on self-reported car driving ability was investigated in two studies by Elliott and colleagues (1997; 2000). In both studies, groups of age-matched participants who underwent FES or SES completed the ADVS, as did a group of age-matched healthy controls. The time between testing was also similar between the surgery groups and shorter in the control group. Both studies found significant improvements in both day and night driving for both surgeries. Furthermore, the percentage of participants returning the highest possible score returned to the same level as the healthy controls for day driving following both surgeries and for night driving following SES. This demonstrates two things, firstly, that cataract surgery has a largely positive effect on self-reported driving ability and, secondly, that strong ceiling effects are observed when using the ADVS to measure self-reported driving ability implying that other measures may be better suited to assessing changes in self-reported driving ability.

##### 3.2.2.1 Conclusion

Two studies presented here used the VF-14 to investigate changes to subjective vision-related function following SES, with neither study reporting a significant effect of SES (Castells et al., 1999; Foss et al., 2006). However, Foss and colleagues (2006) failed to report the motor sub-scales separately making interpreting these results regarding motor function specifically, impossible. Foss and colleagues (2006) also collected data on the individuals’ ability to complete activities of daily living but found no significant effect of SES. Similarly, no significant effect of SES was found for fall rates (Foss et al., 2006; Gia To et al., 2014); however, there is no direct comparison made between post-FES and post-SES groups in either of the papers presented here, making the interpretation of the results difficult. Significant changes to overall disability and a trend toward improved driving ability were found when comparing ISBCS and DSBCS surgery groups (when the DSBCS group were post-FES), yet these results are not directly comparable to the other work presented here due to the differences in surgical protocols employed between the groups (Lundstrom et al., 2006). Elliott and colleagues (1997; 2000) robustly showed that both FES and SES had a positive effect on both day and night driving, however, the strong ceiling effects demonstrated the need for better tools to measure changes in this area.

## 4 Discussion

As was set out in the earlier stages of this systematic review, objective measures of motor function will be considered as our primary outcome measures and subjective measures of motor function will be considered as supplementary, supporting evidence.

Regarding the effect of SES on an individual’s mobility, objective measures suggest that SES largely has a positive effect on mobility (Elliott et al., 1997; 2000; Lee et al., 2013) and is associated with a reduction in fall rates (Meuleners et al., 2014). In contrast, the evidence from self-report measures suggests that SES has no significant effect on fall rate, despite a trend toward reduced incidence of falls (Foss et al., 2006; Gia To et al., 2014; Feng et al., 2018). The lack of positive effect on fall rate following SES may be due to three factors. Firstly, when using a within-participant design, participants will be older post-SES when compared to post-FES. Age is a major predictor of fall risk, for example Meuleners and colleagues (2014) found that participants over the age of 85 were 7 times more likely to fall as those aged 60–65, therefore, despite many researchers attempting to mitigate the effect of age on motor function by including age as a factor in statistical analyses (Gia To et al., 2014; Meuleners et al., 2014; Feng et al., 2018), the increased age of the SES patients may still be masking any positive effects SES has on fall risk. Secondly, SES is associated with an increase in physical leisure activity (Feng et al., 2018; Meuleners et al., 2019) which is associated with an increased risk of falls (Chan et al., 2007) which, similar to the effect of age on fall risk, may be masking the positive effect of SES. Thirdly, the extremely large sample size sample size used by Meuleners et al. (2014) will lead to an increase in statistical power and, therefore, increase the opportunity to detect a significant result (Rusticus and Lovato, 2014). In future studies, a summary of when falls occurred may be useful in interpreting the results.

The evidence regarding the effect of SES on driving is also mixed for both objective and subjective measures. Meuleners and colleagues (2021) found strong evidence that SES predicted a reduction in crashes/near crashes and incidences of speeding in a simulated driving study, however, Agrament and colleagues (2018) found SES did not have a significant effect on levels of driver self-regulation. Similarly, despite using the same measure of driving performance, the ADVS, Lee and colleagues (2013) found SES had little effect on either day or night driving, whereas Elliott and colleagues (1997; 2000) found that SES predicted day driving performance returning to the same level as healthy controls (despite this change being non-significant) and a significant improvement in night driving (also to the same level as healthy controls). The results of Lee and colleagues (2013) are, however, particularly difficult to interpret for two reasons. Firstly, the participants are split into three groups, no surgery, unilateral cataract surgery and bilateral cataract surgery, however, the cataract status of the no surgery and unilateral cataract surgery groups are not made clear meaning that there may be some visual deficits in these groups that are not accounted for in the analysis. Secondly, the group sizes are highly heterogeneous with 1,620 in the no surgery group, 90 in the unilateral surgery group and 29 in the bilateral surgery group. Unequal group size may reduce the power of the experiment, impairing the ability to detect an effect (Rusticus and Lovato, 2014). Information was also gathered about the effect of SES on ADLs and specific motor tasks and suggests that some tasks, such as sewing and fine-handwork and using a personal computer are significantly improved following SES (Akman et al., 2019), however, there was no significant effect of SES on an individual’s ability to perform ADLs such as cooking and writing (Castells et al., 1999).

The present work builds on a review published in 2013 investigating the benefits of SES for older adults, these benefits included changes to vision, quality of life, fall risk/mobility and driving performance (Ishikawa et al., 2013). Overlaps can be drawn with the current review in the evaluation of the quality of life, fall risk/mobility and driving performance. Ishiwaka and colleagues (2013) rated the evidence for improved quality of life as mixed and for fall risk/mobility and driving performance as limited. However, the present review presents mixed evidence for all three groups of motor function: quality of life (ability to perform ADLs), fall risk/mobility and driving performance. These differences are most likely due to the increased number of studies assessing motor function in the present study, in fact, 8 of the 13 studies in the present review were published after the literature search conducted by Ishiwaka and colleagues was completed (9th January 2013). The time lag between the publication of these two reports has seen particularly strong growth in the assessment of the effect of SES on driving performance. To this end, we now have a much better understanding of the benefits of SES in reducing crashes/near crashes and speeding (Meuleners et al., 2021), although, there is still a need for further research in this area, whereby driving simulation seems to be a particularly powerful tool to measure changes to driving performance in a controlled, repeatable and safe manner following SES, as suggested in a 2016 review of cataract and driving performance (Agramunt et al., 2016).

Of the studies included in this review, two key limitations have been identified. Firstly, there is a tendency to use pre-FES participants as the reference point for statistical analysis, whilst excluding direct comparisons between post-FES and post-SES performance levels, a practice that can be seen in 4 of the 11 studies included in this systematic review (Gia To et al., 2014; Meuleners et al., 2014; Agramunt et al., 2018; Feng et al., 2018). Secondly, in the study by Lee and colleagues (2013) the cataract history of the control group is not specified, they are simply referred to as having not received cataract surgery, thus limiting the ability of the reader to draw meaningful conclusions about the benefits of cataract surgery. To a lesser extent, the reporting of inter-surgery and inter-testing intervals could be made explicit to reassure the reader that participants have had similar opportunities for recovery.

The results of this systematic review and the noted limitations of the included studies have several implications for further research. Firstly, the impact of SES on driving and ADLs remains unclear and needs to be further established. Secondly, the inclusion of a well-defined, healthy, aged-matched control group appears useful in drawing firm conclusions about the benefits of SES. Thirdly, direct statistical comparisons must be made between the performance of post-FES and post-SES groups. More broadly, to begin to draw powerful and impactful conclusions about the effect of SES on the motor system there must be a greater degree of agreement on the best practice for testing these constructs. These changes are essential to maintain the high standards of experimental rigour needed to guide policy regarding access to SES. An issue that, in an ageing population, stands to impact many people’s lives and deserves to be based on high-quality research, rather than the whim of the policymakers in any given area.

## Data Availability

The original contributions presented in the study are included in the article/Supplementary Material, further inquiries can be directed to the corresponding author.
