# Supplementary material for: A Systematic Review of the Effects of Second-Eye Cataract Surgery on Motor Function
Source: Front Aging. 2022 Jun 22;3:866823. doi: 10.3389/fragi.2022.866823 (PMC9261376; doi:10.3389/fragi.2022.866823)
Supplement: Supplementary file 1 [file Table1.DOCX]

***Supplementary Material***

1. cataract.mp. [mp=ti, ab, hw, tn, ot, dm, mf, dv, kw, fx, dq, nm, kf, ox, px, rx, ui, sy, tc, id, tm, mh]

2. (surg* or removal or extract*).mp. [mp=ti, ab, hw, tn, ot, dm, mf, dv, kw, fx, dq, nm, kf, ox, px, rx, ui, sy, tc, id, tm, mh]

3. (first?eye or single?eye or unilateral or monocular or second?eye or bilateral or binocular).mp. [mp=ti, ab, hw, tn, ot, dm, mf, dv, kw, fx, dq, nm, kf, ox, px, rx, ui, sy, tc, id, tm, mh]

4. (motor?function or motor?control or mobility or fall* or driv* or daily?living or physical or activ*).mp. [mp=ti, ab, hw, tn, ot, dm, mf, dv, kw, fx, dq, nm, kf, ox, px, rx, ui, sy, tc, id, tm, mh]

5. (child* or congenital).mp. [mp=ti, ab, hw, tn, ot, dm, mf, dv, kw, fx, dq, nm, kf, ox, px, rx, ui, sy, tc, id, tm, mh]

**6. (1 and 2 and 3 and 4) not 5**

**Supplementary Text 1.** Search terms.
